# Transcellular transport of West Nile virus-like particles across human endothelial cells depends on residues 156 and 159 of envelope protein

**DOI:** 10.1186/1471-2180-10-165

**Published:** 2010-06-08

**Authors:** Rie Hasebe, Tadaki Suzuki, Yoshinori Makino, Manabu Igarashi, Satoko Yamanouchi, Akihiko Maeda, Motohiro Horiuchi, Hirofumi Sawa, Takashi Kimura

**Affiliations:** 1 Department of Molecular Pathobiology, Hokkaido University Research Center for Zoonosis Control, Kita 20, Nishi 10, Kita-ku, Sapporo 001-0020, Japan; 2 Laboratory of Veterinary Hygiene, Graduate School of Veterinary Medicine, Hokkaido University, Kita 18, Nishi 9, Kita-ku, Sapporo 060-0818, Japan; 3 Department of Global Epidemiology, Hokkaido University Research Center for Zoonosis Control, Kita 20, Nishi 10, Kita-ku, Sapporo 001-0020, Japan; 4 Global COE program, Hokkaido University, Japan

## Abstract

**Background:**

West Nile virus (WNV) causes viremia after invasion to the hosts by mosquito bite. Endothelial cells could play an important role in WNV spread from the blood stream into the central nervous system and peripheral tissues. Here, we analyzed the capacity of virus-like particles (VLPs) of the highly virulent NY99 6-LP strain (6-LP VLPs) and the low virulence Eg101 strain (Eg VLPs) to cross cultured human endothelial cells.

**Results:**

6-LP VLPs were transported from the apical to basolateral side of endothelial cells, whereas Eg VLPs were hardly transported. The localization of tight junction marker ZO-1 and the integrity of tight junctions were not impaired during the transport of 6-LP VLPs. The transport of 6-LP VLPs was inhibited by treatment with filipin, which prevents the formation of cholesterol-dependent membrane rafts, suggesting the involvement of raft-associated membrane transport. To determine the amino acid residues responsible for the transport of VLPs, we produced mutant VLPs, in which residues of E protein were exchanged between the 6-LP and Eg strains. Double amino acid substitution of the residues 156 and 159 greatly impaired the transport of VLPs.

**Conclusion:**

Our results suggest that a transcellular pathway is associated with 6-LP VLPs transport. We also showed that the combination of the residues 156 and 159 plays an important role in the transport of VLPs across endothelial cells.

## Background

West Nile virus (WNV), a mosquito-borne single-stranded RNA virus, had been known to cause endemic febrile disease in Africa, the Middle East, Europe and Asia [1-4]. Since the concurrent outbreaks of encephalitis among humans, horses and birds in New York in 1999 [5-7], WNV has spread rapidly across North America [8]. WNV has considerable public health impact because of large annual epidemics of human neuroinvasive disease [9]. WNV proliferates in birds and is transmitted to humans, horses and other animals by mosquitoes. After invading the hosts, WNV seems to proliferate in lymphoid tissue and causes viremia [10]. WNV then penetrates the blood brain barrier (BBB) and causes encephalitis with neuronal cell death. Neurons are the main target of the virus in the central nervous system (CNS), since viral antigens are mainly detected in these cells [11].

In addition to the neuronal disease, WNV-associated inflammation outside the CNS can occur in humans. Khouzam [12] reported the case of a patient who had diffuse myocardial damage secondary to WNV infection. Rhabdomyolysis was reported in a patient with WNV encephalitis [13]. Armah *et al*. [14] reported systemic distribution of WNV infection in 6 human cases in which viral antigens were detected in CNS, kidney, lungs, pancreas, thyroid, intestine, stomach, esophagus, bile duct, skin, prostate and testis. These studies suggest that WNV can invade and proliferate in multiple tissues.

Shirato *et al*. [15] suggested that the difference in the neuroinvasiveness between the highly virulent NY99 strain and the non-lethal Eg 101 (Eg) strain is associated with the viral replication in spleen. One of the reasons NY99 strain gains this virulent phenotype might be an enhancement of invasiveness to the peripheral tissues. Blood-borne pathogens must encounter endothelial cells of blood capillaries to invade the target organs. Verma *et al*. [16] demonstrated the mechanism by which WNV crosses endothelial cells using human brain microvascular endothelial (HBMVE) cell culture. Their data suggested that WNV crosses HBMVE cells via a transcellular pathway after viral replication in endothelial cells. However, the possibility that WNV crosses endothelial cells without viral replication cannot be excluded, since WNV infection of endothelial cells is rarely detected in human cases [17]. It is still unclear if a transcellular mechanism is also involved in viral invasion to endothelial cells of peripheral tissues.

In this study, we assessed the possibility that WNV has an ability to cross human endothelial cells. To eliminate the influence of viral replication in endothelial cells, we used virus-like particles (VLPs) which can infect susceptible cells without production of progeny virions. Our results suggest that VLPs of the NY99-6922 6-LP (6-LP) strain cross human umbilical vein endothelial cells (HUVEC) by a transcellular pathway. We also showed that the 6-LP VLPs were transported greater than VLPs of the low-virulence Eg strain, which depends on Ser 156 and Val 159 of E protein.

## Results

### WNV 6-LP VLPs are transferred across human endothelial cells

HUVEC were seeded on the membranes of transwells, which have 0.4 μm pores. The presence of the tight junction with an increase of transendothelial electrical resistance (TEER; 66-77 Ωcm^2^) was confirmed 3 days after seeding. Here we used VLPs previously reported by Scholle *et al*. [18]. VLPs can infect cells because of the presence of the structural proteins (C, prM/M and E protein) that are present in infectious virions. VLPs contain replicon RNA, which encodes the WNV nonstructural proteins and the enhanced green fluorescent protein (eGFP), but lacks the sequence of structural proteins. After VLP infection of susceptible cells, replicon RNA is released and replicates in the cytoplasm accompanied by the expression of eGFP. However, progeny particles are not produced because of the lack of expression of structural proteins in VLP-infected cells.

**Figure 1:**
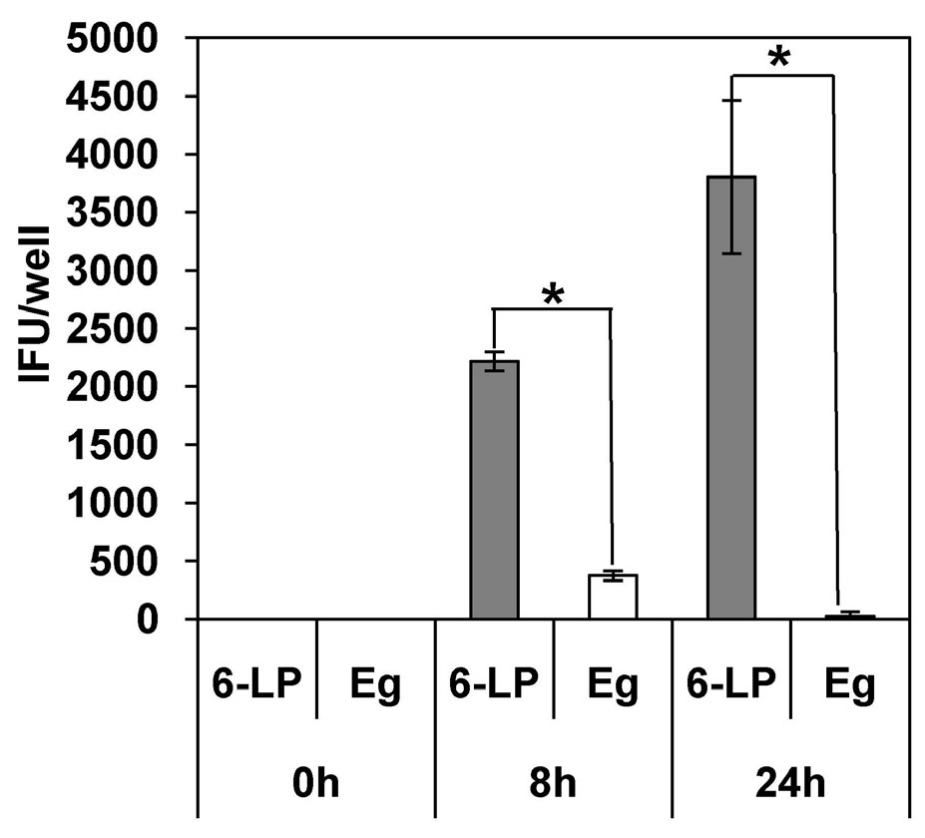
**Transport of 6-LP and Eg VLPs across a monolayer of HUVEC**. HUVEC were exposed to VLPs for 0, 8 or 24 h. The numbers of transferred VLPs were determined by IFU assay. Gray bars, 6-LP VLPs. White bars, Eg VLPs. The graphs show the mean of three determinations. The error bars show SD. The results are representative of 2 independent experiments. *p < 0.01.

To assess the possibility that HUVEC can transport VLPs, HUVEC were exposed to 6-LP VLPs or Eg VLPs at a multiplicity of infection (m.o.i.) of 2 (4 × 10^4 ^infectious unit/transwell). The number of VLPs transferred to the lower chambers was determined by infectious unit (IFU) assay at 0, 8 and 24 h post infection (p.i.) (Fig. 1). 6-LP VLPs were detected at 8 h p.i. and increased approximately 2-fold at 24 h p.i. On the other hand, few Eg VLPs were detected at 8 and 24 h p.i. The amount of the transferred 6-LP VLPs was significantly higher than that of Eg VLPs at 8 and 24 h p.i. (p < 0.01). These results suggested that 6-LP VLPs were transferred across HUVEC and that the transfer of Eg VLPs was much less efficient.

### 6-LP VLPs were transported without altering the integrity of tight junction

Verma *et al*. [16] suggested that WNV replicates in the HBMVE cells and that the progeny virus crosses the BBB via a transcellular pathway without impairing the integrity of tight junction. However, VLPs used in this study do not produce progeny virions. Thus, there is a possibility that 6-LP VLPs cross from the apical to the basolateral side by disrupting tight junction.

**Figure 2:**
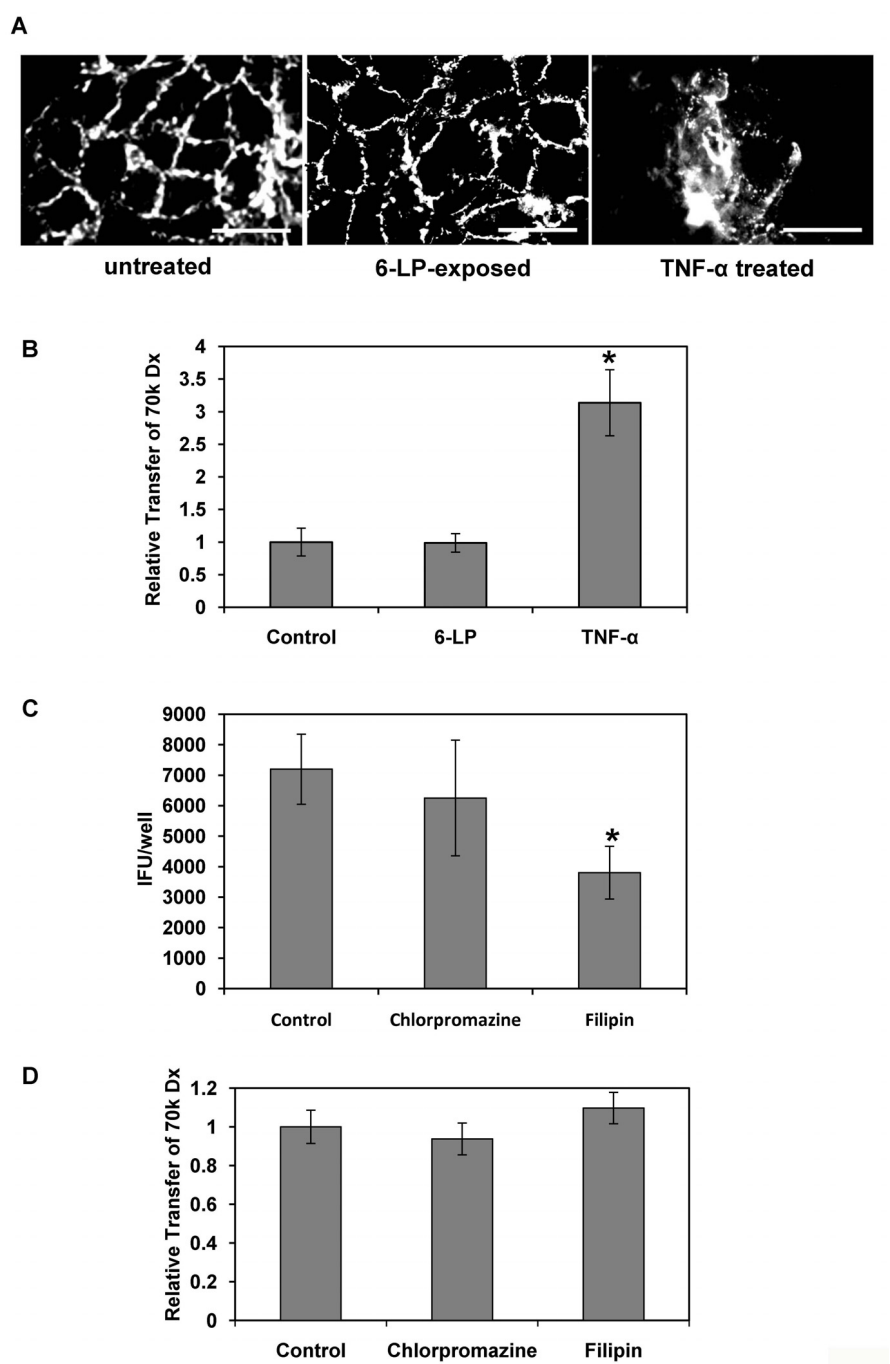
**Transcellular transport of 6-LP VLPs in HUVEC**. (A) Distribution of tight junction marker ZO-1 in HUVEC. HUVEC were exposed to 6-LP VLPs or treated with TNF-α for 24 h. The cells were fixed and processed for immunofluorescence staining of ZO-1. Bars represent 50 μm. (B) Transfer of Dx70k into a monolayer of untreated, 6-LP VLP-exposed or TNF-α treated HUVEC. HUVEC were exposed to 6-LP VLPs or treated with TNF-α in the presence of FITC-labeled 70k Dx (FITC-70k Dx). After 24 h, media were collected from lower chambers and the fluorescence of transferred 70k Dx was measured by a fluorescent plate reader. Relative transfer of FITC-70k Dx was calculated as described in METHODS. The graphs show the mean of three determinations. The error bars show SD. The results are representative of 2 independent experiments. *p < 0.01. (C) Transport of 6-LP VLPs in HUVEC treated with endocytosis inhibitors. HUVEC were exposed to 6-LP VLPs in the presence or absence of 5 μg/ml of chlorpromazine or 1 μg/ml of filipin. The cells treated with 0.1% DMSO were used as control. After 24 h, media at the lower chamber were collected and subjected to IFU assay. *p < 0.01. (D) Transfer of FITC-70k Dx in HUVEC treated with endocytosis inhibitors. FITC-70k Dx was added to HUVEC with or without 5 μg/ml of chlorpromazine or 1 μg/ml of filipin. After 24 h, medium was collected from the lower chambers and the fluorescence was measured. Relative transfer of FITC-70k Dx was calculated as described in METHODS. The graphs show the mean of three determinations. The error bars show SD. The results are representative of 2 independent experiments.

To assess this possibility, the distribution of a tight junction marker ZO-1 was analyzed by immunocytochemistry at 24 h p.i. (Fig. 2A). The localization of ZO-1 was not visibly affected in 6-LP VLP-exposed HUVEC, when compared to the untreated control. We also measured the permeability of 70k Dextran (Dx) to check the integrity of the tight junction (Fig. 2B). The permeability of 70k Dx in VLP-exposed HUVEC was similar to that in untreated cells by this assay. It has been known that TNF-α exposure induces changes in endothelial cell morphology and permeability [19]. Therefore, we treated the cells by TNF-α as a control. Treatment of HUVEC with TNF-α at 2 μg/ml greatly impaired the integrity of the tight junction (p < 0.01; Figs. 2A and 2B).

### 6-LP VLPs cross HUVEC via a transcellular pathway

To assess the involvement of a transcellular pathway, we examined the effects of chlorpromazine and filipin on VLP transport. Chlorpromazine disrupts the recycling of AP-2 from endosomes and prevents the assembly of clathrin-coated pits on the plasma membrane [20]. Filipin is a sterol-binding agent and prevents the formation of cholesterol-dependent membrane rafts [21]. The optimal concentration of chlorpromazine and filipin was determined by the inhibition of the uptake of transferrin and cholera toxin subunit B, which are known as ligands for clathrin-and lipid-rafts-dependent endocytosis, respectively (data not shown). HUVEC were exposed to 6-LP VLPs in the presence or absence of the inhibitor. FITC-labeled 70k Dx was also added to the transwells with 6-LP VLPs to evaluate the tight junction integrity. The transport of VLPs was inhibited by filipin (p < 0.01), but was not significantly by chlorpromazine (Fig. 2C). In contrast, the permeability of 70k Dx was not impaired by either chlorpromazine or filipin (Fig. 2D). These results suggested that lipid rafts are involved in VLPs transport.

### Transport of 6-LP VLPs depends on E protein

**Figure 3:**
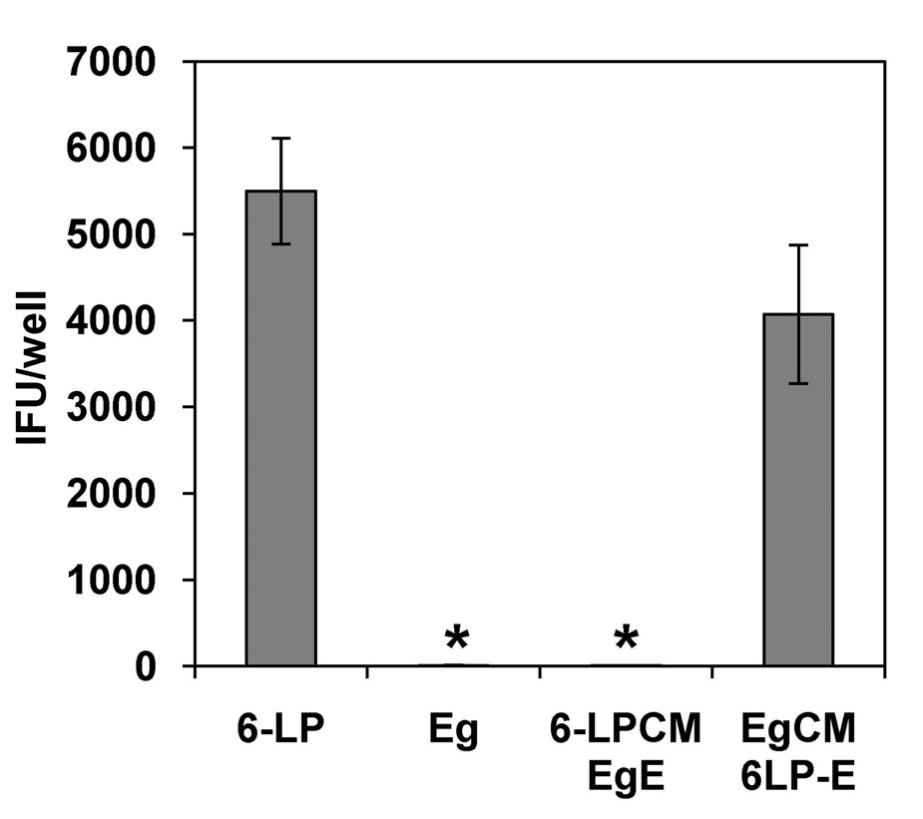
**Role of WNV E protein in the transport of VLPs. HUVEC were exposed to 6-LP, Eg, 6-LP CM Eg E or Eg CM 6-LP E VLPs**. After 24 h, media at the lower chamber were collected and subjected to IFU assay. The graphs show the mean of three determinations. The error bars show SD. The results are representative of 2 independent experiments. * represents p < 0.01 (versus 6-LP).

It is known that E protein interacts with viral receptors on the host cells [22-28] resulting in the induction of receptor mediated endocytosis [25,29,30]. To examine whether E protein is involved in the transport of VLPs, we generated chimeric VLPs using 6-LP and Eg VLPs. 6-LP CM Eg E VLPs have C and M/prM proteins derived from 6-LP strain and E protein from Eg strain. Eg CM 6-LP E VLPs have C and M/prM protein from Eg strain and E protein from 6-LP strain. HUVEC were exposed to wild type or chimeric VLPs and transported VLPs were detected by IFU assay at 24 h p.i (Fig. 3). The transport of Eg CM 6-LP E VLPs was similar to that of wild type 6-LP VLPs and was significantly higher than those of 6-LP CM Eg E VLPs and wild type Eg VLPs (p < 0.01). 6-LP CM Eg E VLPs were rarely transported across HUVEC as well as wild type Eg VLPs. These results suggest that the transport of VLPs across HUVEC is strongly affected by E protein.

### Multiple amino acid residues of E protein influence the transport of 6-LP VLPs

**Figure 4:**
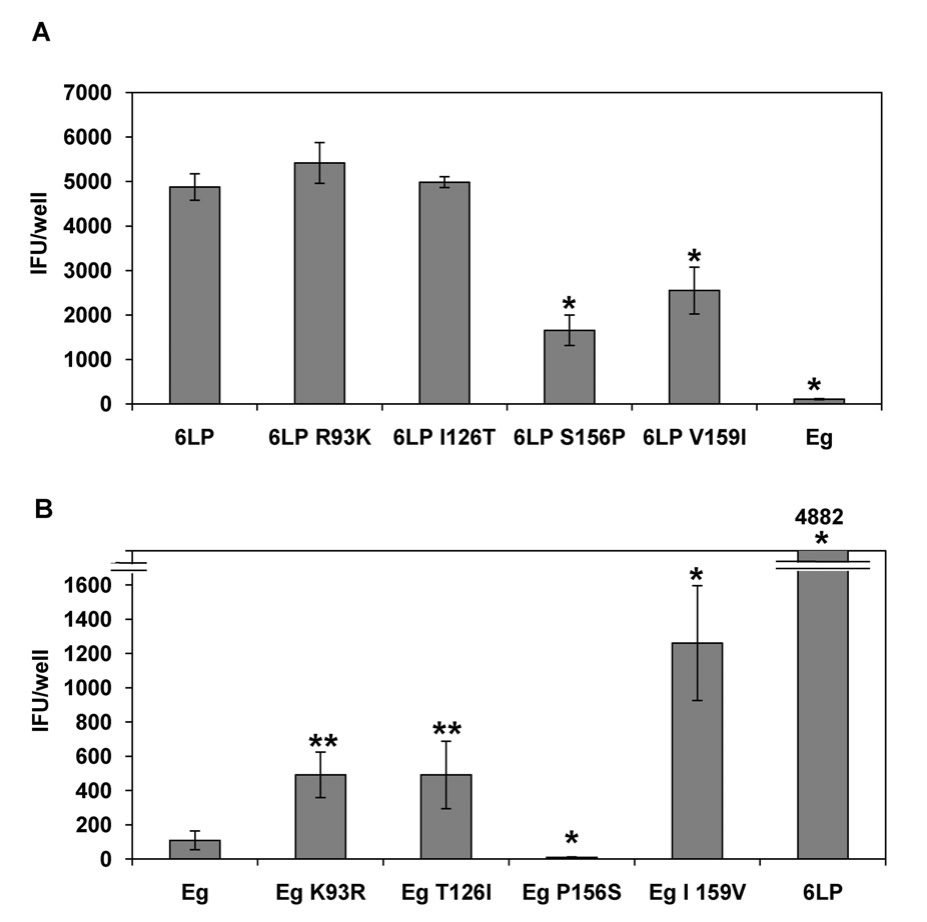
**Effect of single amino acid substitutions in E protein on the transport of VLPs**. HUVEC were exposed to mutant VLPs. After 24 h, media at the lower chamber were collected and subjected to IFU assay. (A) Transport of mutant 6-LP VLPs. *represents p < 0.01 (versus 6-LP). (B) Transport of mutant Eg VLPs. * and ** represent p < 0.01 and p < 0.05, respectively (versus Eg). The graphs show the mean of three determinations. The error bars show SD. The results are representative of 2 independent experiments.

The E proteins of the 6-LP and Eg strain differ at 4 amino acid residues. To determine the residues that enhance the transport of 6-LP VLPs, we produced mutant VLPs (Table 1). 6-LP S156P VLPs and 6-LP V159I VLPs had significantly reduced transport compared to wild type 6-LP VLPs (p < 0.01) although the amount of transported VLPs was much higher than that of Eg VLPs (p < 0.01; Fig. 4A). As shown in Fig. 4B, Eg K93R VLPs and Eg T126I VLPs showed increased transport compared to wild type Eg VLPs (p < 0.05). The transport of Eg I159V was significantly increased (p < 0.01), although it was much lower than 6-LP VLPs. Previous studies reported that Ser 156 is involved in the N-linked glycosylation at 154, which is important for virulence and neuroinvasion [31-34]. Therefore, we expected that the transport of Eg P156 S would be increased. However, the transport of Eg P156 S VLPs was significantly lower than that of WT Eg VLPs (p < 0.01). These results suggest that multiple residues of E protein can influence the transport of VLPs.

**Table 1:** Single and double mutant VLPs

Name	Wild type	Position^1^	Substitution^2^
6-LP R93K	6-LP	93	R→K
6-LP I126T	6-LP	126	I→T
6-LP S156P	6-LP	156	S→P
6-LP V159I	6-LP	159	V→I
Eg K93R	Eg	93	K→R
Eg T126I	Eg	126	T→I
Eg P156S	Eg	156	P→S
Eg I159V	Eg	159	I→V
6-LP S156P V159I	6-LP	156, 159	S→P, V→I
Eg P156 S I159V	Eg	156, 159	P→S, I→V

^1 ^Amino acid position of E protein.

^2 ^Amino acid substitution from wild type to mutant.

### The combination of Ser 156 and Val 159 is important for the transport of 6-LP VLPs

**Figure 5:**
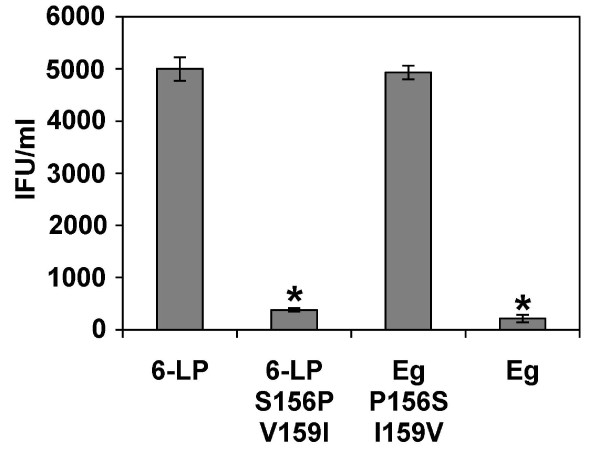
**Effect of double amino acid substitutions of E protein on the transport of VLPs**. HUVEC were exposed to 6-LP, 6-LP S156P V159I, Eg P156 S I159V or Eg VLPs. After 24 h, media at the lower chamber were collected and subjected to IFU assay. * p < 0.01 (versus 6-LP). The graphs show the mean of three determinations. The error bars show SD. The results are representative of 2 independent experiments.

From the result of Fig 4B, the transport of Eg P156 S did not increase. This finding suggests the possibility that the combination of amino acids at the position of 156 and 159 might affect the transport of VLPs. To assess this hypothesis, we generated double mutants, 6-LP S156P V159I and Eg P156 S I159V (Table 1). As shown in Fig. 5, the transport of 6-LP S156P V159I was greatly reduced (p < 0.01; versus 6-LP VLPs) to the level of wild type Eg VLPs. The transport of Eg P156 S I159V was greatly increased (p < 0.01; versus Eg VLPs) to the level of wild type 6-LP VLPs. These results suggest that the combination of Ser 156 and Val 159 is important for the transport of 6-LP VLPs across HUVEC.

### Combination of amino acid sequence at 156 and 159 does not affect the N-linked glycosylation of E protein

**Figure 6:**
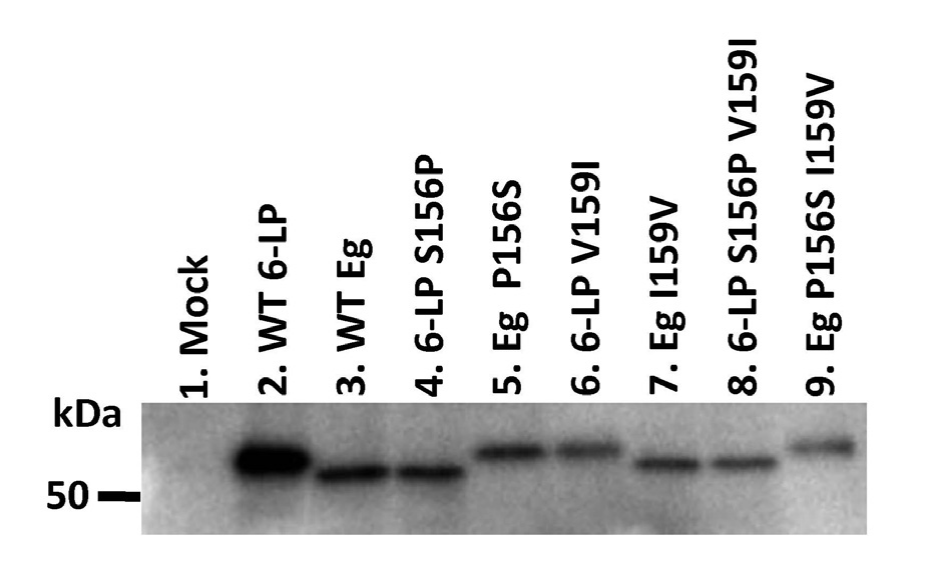
**Glycosylation of E protein in wild type and mutant VLPs**. 293T cells were cotransfected with replicon RNA and plasmids encoding structural genes or empty vector for mock control. The supernatants were collected and subjected to Western blotting with anti-WNV E protein monoclonal antibody.

From the results of Figs. 4 and 5, we speculated that the combination of amino acid sequence at 156 and 159 might affect N-linked glycosylation at the position 154 resulting in unglycosylation of E protein of Eg P156 S. To assess this possibility, we analyzed the glycosylation of E protein in 6-LP VLPs, Eg VLPs, 6-LP S156P, Eg P156 S, 6-LP V159I, Eg I159V, 6-LP S156P V159I and Eg P156 S I159V. Western blotting of E protein showed the band of wild type 6-LP strain was higher than that of Eg strain (Fig. 6. lanes 2 and 3) because of glycosylation. E protein of 6-LP S156P, Eg I159V and 6-LP S156P V159I was unglycosylated (Fig. 6. lanes 4, 7 and 8), whereas E protein of 6-LP V159I and Eg P156 S I159V was glycosylated (Fig. 6. lanes 6 and 9). Interestingly, E protein of Eg P156 S was also glycosylated (Fig. 6. lane 5). These results suggest that the combination of the residues 156 and 159 does not affect the N-linked glycosylation and that glycosylation of E protein is not the determinant of the transport of VLPs.

## Discussion

WNV NY strains have a highly virulent phenotype compared to the Eg strain which was isolated in Africa. Their enhanced replication in peripheral tissues may lead to long-lasting viremia resulting in increasing incidence of viral invasion to CNS. The interaction of the virus with endothelial cells of blood capillaries could be involved in WNV invasion to target organs. In this study, we assessed the transport of WNV NY99 6-LP strain and Eg strain across human endothelial cells. Our data demonstrate that VLPs of the 6-LP strain were transported across human endothelial cells more than VLPs of the Eg strain.

Microbial invasion across endothelial cells can occur through transcellular pathway mediated by vesicles, paracellular entry after disruption of the tight junctions, or "Trojan horse" mechanism by transport within circulating phagocytic cells [35,36]. Our data indicate that 6-LP VLPs are transported by a transcellular pathway, because the transport of VLPs was inhibited by the treatment with filipin, a modifier of lipid raft-associated membrane transport. Clathrin-dependent pathways seem to be less important because the treatment with chlorpromazine had no significant effect on the transport of VLPs. Paracellular entry is unlikely to be involved in transport of VLPs because the structure of ZO-1 and the permeability of Dx 70k were not altered during VLP transport.

Our data partially support the results by Verma *et al*. [16] which suggested that WNV crosses HBMVE cells without altering the integrity of tight junction. The authors concluded that WNV replicates in endothelial cells and the progeny viruses are transported from the apical to basolateral side. However, our data suggest that WNV can be transported across endothelial cells without viral replication. Cell type difference could be the most reasonable explanation, because several studies showed that there are differences between HBMVE cells and HUVEC in the production of growth factors, immunoregulatory factors and adhesion molecules [37-39]. HBMVE cells and HUVEC differentially respond to cytokine treatment resulting in the different cytokine production and leukocyte recruitment [40,41]. Particularly, modulation of adhesion molecules can affect endocytosis [37]. Therefore, our data seem to reflect events that can occur in peripheral tissues having tight junction such as heart and muscles rather than in CNS.

In WNV-infected mice, viral replication in peripheral tissues results in the inflammatory cytokine production such as TNF-α, IL-6 and macrophage migration inhibitory factor [42-45]. Although the role of these cytokines in infection still remains controversial, vascular permeability can be affected by the presence of these cytokines [45]. One of the mechanisms for the impairment of vascular permeability by these cytokines is disruption of tight junctions of endothelial cells [46]. Promotion of vesicular transport of endothelial cells, including pinocytosis and transcytosis, is also affected by these cytokines [47]. Paracellular invasion by disruption of the tight junction induced by cytokines could occur in vivo, however, there is a possibility that WNV also utilizes a transcellular pathway, which might be promoted by inflammatory cytokines.

The analysis of VLPs with chimeric E proteins showed that E protein determines the difference in the transport across HUVEC between the 6-LP and Eg strains. Our data also suggest that multiple amino acid residues of E protein are influential. It has been well known that the sequence NYS/T at the residues 154-156 is important for glycosylation associated with the virulence of WNV and that strains possessing proline at the residue 156 lack glycosylation [10,31-33]. The prototype WNV strain B956 has a 4 amino acid deletion in the residues 156-159 resulting in absence of glycosylation [48]. The position of glycosylation seems to be also important, since the WNI-25 and WNI-25A strains which have N-glycosylation at the residue 155, do not show neuroinvasive phenotype [49,50]. The present study suggests that the combination of Ser 156 and Val 159 is important for transport of VLPs across endothelial cells, which might be associated with the invasion of WNV into the target organs.

The transport of Eg P156 S VLPs was lower than that of WT Eg VLPs in spite of the presence of glycosylation. The residues 156-160 form two turns of α-helix, termed αA', although E proteins of Dengue virus serotype 2 (DENV-2) and Tick-borne encephalitis virus (TBEV) lack the amino acids 157-160 resulting in the absence of this structure[51]. The α-helix shifts the glycosylation site about 5 Å to the exterior and lateral surfaces of E protein with respect to those of E proteins of DENV-2 and TBEV. Davis *et al*. [52] showed that modulation of N-glycosylation of WNV E protein modified the attachment to DC-SIGNR. As well as the existence of proline and the deletion of the amino acids between the residues 156-160, there is a possibility that the combination of amino acid residues at 156 and 159 might affect the structure of αA' and position of glycosylation site, resulting in modulation of the binding affinity to a lectin or unknown binding molecules on HUVEC. This, in turn, could be a reason for the unsuccessful transport of Eg P156 S VLPs.

## Conclusion

In this study, we propose a transcellular mechanism by which WNV crosses endothelial cells and enters the target organs. We also suggest that higher transendothelial migration ability could be one of the determinants of the different virulence of the NY and Eg strains, and that this depends on Ser 156 and Val 159 of E protein.

## Methods

### Cell culture

HUVEC were purchased from Lonza Group Ltd. and cultured in EGM-2 Endothelial Cell Growth Medium-2 supplemented with SingleQuots growth factors, cytokines and supplements (Lonza). The cells at passage 5 were used for experiments. Vero cells were cultured in Eagle's minimum essential medium (MEM; Nissui, Tokyo, Japan) supplemented with 5% fetal bovine serum (FBS; Sigma). Baby hamster kidney (BHK) cells were cultured in MEM supplemented with 10% FBS. HEK293T cells were cultured in Dulbecco's Modified Eagle Medium (Nissui).

### Plasmid Constructs

The WNV 6-LP and Eg strains were provided by Dr. I. Takashima, Hokkaido University, Japan [15,34]. 6-LP strain was established by plaque purification from WNV NY99-6922 strain, which was isolated from mosquitoes in 1999 [34]. Complement DNA (cDNA) of the structural genes (C, prM/M and E) of the 6-LP and Eg strains were prepared by RT-PCR and subcloned into pCXSN, which was generated from pCMV-Myc (Takara Bio, Shiga, Japan) by replacing the sequence of the Myc tag and multicloning site with restriction enzyme sites of *Xho *I, *Sal *I and *Not *I. The resultant plasmids were designated pCXSN 6-LP CME and pCXSN Eg CME, respectively. For the construction of chimeric VLPs between 6-LP and Eg, a *Sma *I site was generated by substitution of t to c (in 6-LP) and a to g (in 6-LP and Eg) at nucleotide positions 460 and 463, respectively, of the prM gene by PCR. The sequence containing the prM gene (nucleotides 461-555) and E gene (nucleotides 1-1500) was digested by *Sma *I and *Not *I from pCXSN 6-LP CME or pCXSN Eg CME and inserted into pCXSN Eg CME or pCXSN 6-LP CME. The resultant plasmids were designated pCXSN Eg CM 6-LP E and pCXSN 6-LP CM Eg E, respectively. The constructs for single or double mutant VLPs were generated by PCR with pCXSN 6-LP CME or pCXSN Eg CME.

### VLP preparation

WNV replicon cDNA construct (pWNR NS1-5 EG2 AN), was generously provided by Dr. Peter W. Mason, University of Texas Medical Branch, USA [18]. WNVR NS1-5 EG2 AN encodes the nonstructural proteins (NS1-5) of WNV 3356 strain isolated from American crow in 2000 [53], eGFP, autocatalytic foot-and mouth disease virus 2A protease and neomycin phosphotransferase II under the translational control of encephalomyocarditis virus internal ribosomal entry site. One μg of pWNR NS1-5 EG2 AN was linearized with *Xba *I and purified with a PCR purification kit (QIAGEN Inc), followed by ethanol precipitation. WNV replicon RNA was produced with in vitro transcription with an mMESSAGE mMASHINE T7 kit (Applied Biosystems) according to the manufacture's instructions. BHK cells (5 × 10^6^) were trypsinized, washed three times with phosphate-buffered saline (PBS) and resuspended in 450 μl of PBS. Then, 5 μg of replicon RNA was added to the cell suspension and introduced by using a GenePulser II elecroporation apparatus (Bio-Rad Laboratories) at 750 V, 25 μF with the resistance set to ∞. Cells were cultured in 10 cm dishes with MEM supplemented with 10% FBS for 24 h. The culture media were replaced with Opti-MEM (Invitrogen) and incubated at 37°C for 30 min. The cells were transfected with 12 μg of the plasmid encoding the sequence of WNV structural genes by Lipofectamine (Invitrogen). After 48 h, supernatants were collected and cell debris was removed by centrifugation at 1000 g for 5 min. The supernatants were concentrated with Centriplus (Millipore). For the IFU assay, Vero cells in 24 well plates were infected with serial 10-fold dilutions of VLP preparations. After a 1 h incubation at 37°C, the solutions were removed and replaced with the culture media. After 48 h p.i., the number of VLPs-infected cells was counted by eGFP signals and the IFU value was calculated.

### Monolayer cultures of HUVEC and transport assay of VLPs

HUVEC were seeded in transwell inserts for 24 well plates with polycarbonate membranes having 0.4 μm pores (Millipore). The media volumes were 200 μl for transwells and 700 μl for the lower chambers, respectively. The cells were cultured for 3 days and the integrity of tight junctions was evaluated by measuring TEER using a Millicell ERS (Millipore). The wells showing TEER elevation (more than 66 Ωcm^2^) were used for experiments. For VLPs transport assay, HUVEC were exposed to 4 × 10^4 ^IFU/transwell of VLPs (2 m.o.i.). The media in the lower chambers were collected at the indicated time points and subjected to the IFU assay on Vero cells.

### Immunofluorescence of ZO-1

HUVEC seeded in transwells were exposed with 6-LP VLPs or treated with TNF-α. After 24 h, the cells were washed with PBS once and fixed with 4% paraformaldehyde (PFA) in PBS for 10 min at room temperature. After washing with PBS three times, the cells were permeabilized with 0.1% Triton X-100 in PBS and blocked with 2% bovine serum albumin in PBS (blocking solution) for 15 min at room temperature. The primary antibody incubation was performed overnight at 4°C with rabbit antiserum to human ZO-1 (BD Transduction Laboratories) diluted at 1:1000 in blocking solution. Then the cells were washed with PBS three times, and Alexa 488 conjugated donkey anti-rabbit IgG antibodies (Invitrogen) were added at 1:1000 dilution in blocking solution for a 1 h incubation at room temperature. After a PBS wash, the membranes were cut from transwell, placed on cover glasses and observed by fluorescent microscopy.

### 70k Dextran transfer assay

Fluorescein (FITC)-labeled 70k Dx (Invitrogen) was added into HUVEC with 6-LP VLPs, TNF-α (positive control) or media (negative control). After 24 h incubation at 37°C, 100 μl of medium was collected from each well and transferred into a 96-well plate. The FITC signal was read by a fluorescent plate reader, Mithras LB940 (Berthold). The relative transfer of 70k Dx was calculated by dividing the FITC signal of samples incubated with 6-LP VLPs or TNF-α by the mean of the signal of the negative control. The relative transfer of 70k Dx in the negative control was defined as 1.

### Effect of endocytosis inhibitors on the transport of 6-LP VLPs

For stock solutions, chlorpromazine (Sigma) and filipin III (Sigma) were dissolved in dimethyl sulfoxide (DMSO) at 5 and 1 mg/ml, respectively. HUVEC in transwells were preincubated with the inhibitor at the indicated concentration for 30 min, and exposed to 6-LP VLPs for 24 h. For the control, DMSO was added in the media at concentration of 0.1%. The evaluation of the transported VLPs was performed as described above. The integrity of monolayer of HUVEC was confirmed by the 70k Dx transfer assay described above.

### Western blotting for E protein

Wild type or mutant VLPs were produced with 293T cells as described above. Supernatants from cell cultures were subjected to sodium dodecyl sulfate-polyacrylamide gel electrophoresis and Western blotting with a mouse monoclonal antibody to WNV E protein clone 3.91 D (Millipore) for the primary antibody and horseradish peroxidase (HRP)-conjugated goat antibodies to mouse immunoglobulin (1:5,000 dilution; Biosource). The immunocomplex was visualized with Immobilon™ Western chemiluminescent HRP substrate (Millipore) and LAS-1000 mini (FIJIFILM, Tokyo, Japan).

### Statistical analysis

Quantitative data are expressed as means ± standard deviation (SD) and were compared with Student's *t *test.

## Authors' contributions

Conception and design: RH; Acquisition of data: RH, TS, SY; Analysis and Interpretation of data: RH, TS, YM, MI, AM, MH, HS, TK; Drafting the paper: RH

All authors read and approved the final manuscript.
